# Factors that influence the turnover intention of Chinese village doctors based on the investigation results of Xiangyang City in Hubei Province

**DOI:** 10.1186/s12939-014-0084-4

**Published:** 2014-11-04

**Authors:** Pengqian Fang, Xiangli Liu, Lingxiao Huang, Xiaoyan Zhang, Zi Fang

**Affiliations:** School of Health and Medicine Management, Tongji Medical College, Huazhong University of Science and Technology, 13 Hangkong Road, Qiaokou District Wuhan, 430030 China; Chongqing University of Medical Sciences, Chongqing, China; The London School of Economics and Political Science, PO Box13420, Houghton Street, London, WC2A 2AE UK

**Keywords:** China, Village doctor, Turnover intention, Job satisfaction

## Abstract

**Introduction:**

This study analyzes the factors that influence the turnover intention of village doctors by investigating village clinic workers in rural areas, particularly in Xiangyang City, Hubei Province.

**Methods:**

A total of 1184 village clinics were sampled randomly in Xiangyang City. The research assistants distributed 1930 questionnaires to village doctors. This study had a response rate of 97.88%. A total of 1889 village doctors completed the questionnaires.

**Results:**

The results of the investigation conducted in Xiangyang City indicated that 63.2% of the village doctors did not plan to leave the organization where they were currently employed. However, more than one-third (36.8%) of the village doctors considered leaving their posts voluntarily. Some job satisfaction indexes affect their intention to resign. The results showed that *income satisfaction and the way organization policies are put into practice, in addition, my pay and the amount of work I do, the chances for advancement on this job* and *the work conditions* are significant factors that contribute to the turnover intention of village doctors.

**Conclusions:**

This study may interest heath care management administrator and highlight the influence of job satisfaction on turnover intention of village doctors. Our findings outline some issues that contribute to these problems and suggest an approach for health care policy maker to implement a broader national process and organizational strategies to improve the job satisfaction and stability of the village doctors.

## Introduction

The system of barefoot doctors was conducted in China in the 1950s to look after the health care of the population in rural areas [1]. At the same time, the quantity of barefoot doctors increased dramatically as they were authorized to provide medical services after a short period of training at a county or community hospital [2]. In the early 1990s, village doctors stepped into the stage of standardized management, the Ministry of Health (MOH) put forward regulations aimed at strengthening post-training for the village doctors, which requires total training time should reach more than two years in order to be qualified. Village doctors are General Practitioners in the rural area of China. They did not receive a sufficient medical education, which usually only lasts for two years in medical knowledge training, similar to a two-year education in community college. Village doctor’s main duty is to provide public health services, general disease diagnosis and treatment to rural residents. The public health services includes health education, the prevention and treatment of infectious diseases (such as respiratory infection), child and maternal care, elderly care consultation, chronic disease (such as diabetes, hypertension) management, etc. Most barefoot doctors practiced a mixture of basic western medicine and Traditional Chinese Medicine (TCM) [3]. The “barefoot doctor” service was initiated by peasants on a collective and mutual aid basis [4]. Barefoot doctors practice medicine, which enabled the system of cooperative health care to embark on the path of rural development employing Chinese characteristics such as the low-cost but high-yield healthcare, therefore, a poor country could achieve major health work [5]. Village doctors, being an essential part of grassroots health human resources are important in the areas that lack medicine or doctors.

With the rapid economic growth and social development, the Ministry of Health started to realize the importance of improving village doctors’ quality. One of the most important regulations, the ‘Village Doctors Practitioners Regulation’ was released by the State Council in 2003, which particularly regulated the certification, practice, training and legal obligations of village doctors, establishing the first set of national regulations for village doctors [3]. After the regulations were published, the number of village doctors quickly decreased to 0.8 million in 2003 [6]. The ‘Village Doctors Practitioners Regulation’ clearly put forward that, only those, who passed the qualifying examination and obtained a certificate issued by competent health administrations departments, could work in village clinics as village doctors.

Currently, the healthcare system of China is facing new challenges, such as the increased healthcare demands and expenditure, inefficient use of healthcare resources, unsatisfying implementation of disease management guidelines, and inadequate healthcare insurance [7], and so on. The Chinese central government passed a landmark program for the health system reform in 2009, which was aimed at improving health care for all citizens by strengthening disease control and the primary care system [8,9]. The new medical reform program calls for the necessity to “build a strong rural health service network at the end; improve rural doctors’ wages; improve rural doctors’ training, increase doctors’ reserve capacity, and gradually promote rural doctors’ ability, help them shift to practicing (assistant) doctors through targeted training”. Issued by the Chinese central government, on July, 2011, “the guidance on strengthening establishment of village doctor team” required local governments to formulate and improve the pension policy of village doctors, to improve the working environment, to regulate medical practice of the village doctor and to enhance medical practice levels of village doctors.

Primary care is at the heart of the health reform in China because we are facing an impending shortage of primary care physicians similar to many western countries and other providers considering the aging population and the increased need for healthcare services [10,11]. The shortage and misdistribution of healthcare workforce are the major obstacles to strengthening public healthcare and the primary health care system to achieve the policy goals of healthcare reform [12]. Human resources are underprovided typically in the rural areas due to various reasons including low salary, overburden, and stress [13]. Therefore, more attention should be paid to the development of the healthcare workforce [14].

Hospitals need stable professional health care teams to ensure the quality of the medical service provided. However, the working conditions of village doctors are far from stable considering the insufficient job security and low income. Thus, identifying the factors that could significantly alleviate the turnover intention among the village doctors and thus ensuring the stability of the medical teams in rural areas have become urgent issues that should be immediately addressed.

Several studies show that the job satisfaction of individual medical worker has a significant effect on the stability of the overall workforce and the quality of the health care delivered. A growing number of physicians are leaving or intending to leave their organizations in China because of insufficient job satisfaction [15].

The earliest study on turnover was the participant determination model constructed by March & Simon in 1958 [16]. Mobley et al. proposed a heuristic model of the employee withdrawal decision process, which was used to study the relationship between job satisfaction and employee turnover [17]. The correlation between job satisfaction and turnover intention was evident in health care, and more recent studies noted that the job satisfaction and organizational commitment of employees are closely inter-related and correlated with turnover intention [18]. Borda and Norman showed that for nurses job satisfaction was the most consistent predictor of the intention to leave [19].

Studies on village doctors’ intention to resign are limited compared with other medical works. Scholars and the government pay more attention to training their practice capacity than to finding out the factors that result in the turnover intention of village doctors.

This study analyzes the factors that influence the turnover intention of village doctors by investigating village clinics workers who serve in Xiangyang City, Hubei Province. This research includes their individual characteristics, degree of job satisfaction, turnover intention, and the relationship between job satisfaction and turnover intention. We aim to suggest concrete areas for improvements to the policy makers, so as to help advance basic health care services development in China.

## Methods

### Sampling

Hubei Province in central China was purposely selected because the area has an average number of doctors per thousand populations, and the economic development and medical development of Hubei at a medium level in China. Xiangyang is a prefecture-level city in northwest Hubei, which is composed of 10 counties. This study employed multistage stratified cluster sampling. Five counties, which include a total of 36 townships, were chosen at the first stage according to the levels of economic development. The 36 townships were then chosen, which consists of 1184 village clinics. A questionnaire survey was administered in all 1184 village clinics in Xiangyang. The investigation was conducted during the period of July and August in 2012. A total of 1930 questionnaires were distributed from which 1889 village doctors responded. The method of 1930 questionnaires survey was one-on-one, face to face questionnaire survey.

### Measuring instruments

A six-page questionnaire was produced, which consists of five parts. Part 1 included the basic socio-demographic information of gender, age, marital status, educational qualification, employment mode, technical position, work seniority, average annual income, and title. An assessment of job satisfaction and turnover intention was included in Part 2. Other parts include learning situation, living and social participation situation, and view on patient conflicts. Adapting the Minnesota Satisfaction Questionnaire based on the actual situation of village doctors in China, we developed 12-item general satisfaction inventories. Through reliability statistics, the Cronbach’s Alpha is 0.922. Village doctors were asked for their job satisfaction with the following aspects: been able to keep busy and fulfilling, level of attention by leaders, the way my boss handles his\her workers, the competence of my manager in making decisions, work stability, the chance to do something that makes use of my abilities, the way organization policies are put into practice, my pay and the amount of work I do, the chances for advancement on this job, the chance to try my own methods of doing the job, work conditions, and the feeling of accomplishment I get from the job. These aspects were evaluated in a five-point (numbered from 0 to 4) scale that ranged from “very dissatisfactory” to “very satisfied”, wherein “0” represents very dissatisfactory, “1” represents dissatisfactory, “2” represents moderate (not too bad), “3” represents satisfactory, and “4” represents very satisfactory. The question “do you intend to leave the current employment?” was asked to each village doctor. They replied with “Yes” or “No”.

### Data analysis

The collected data was entered into EpiData3.1. SPSS18.0 was used for related statistical data analysis. The socio-demographic factors of the investigated village doctors were summarized using a descriptive statistical analysis method. Categorical variables were tested by chi-square test to assess whether the socio-demographic and other factors were related to the turnover intention of village doctors. This study used binary logistic regression to judge significantly related factors that result in the turnover intention of village doctors. The binary logistic regression model uses the turnover intention as dependent variable. The variant is whether the village doctors intend (1) or not intend (0) to leave. The significant variants discovered from the Pearson Chi square statistical test were selected and substituted into the Binary logistic regression model (p < 0.05). The odds ratio (OR) was reported with 95% (CI) where applicable. All tests were conducted at the 5% level of significance.

## Results

### Socio-demographic characteristics of study sampling

A total of 1889 village doctors responded from the 1184 village clinics surveyed in Xiangyang City. About 68.9% of the respondents were male and 31.1% were female. The average age of respondents was 45.54 years, with an average working experience of 23.34 years. In this study, 97.9% of the respondents did not have an undergraduate degree, whereas 2.1% had undergraduate degrees or above. What’s more, 23.1% of the respondents didn’t obtain a practicing certificate and 70.5% of village doctors thought that their income levels were in middle or below in the local.

The socio-demographic characteristics of the study group are shown in Table 1 (n = 1889).

**Table 1:** **Socio-demographic characteristics of village doctors (n = 1889)**

**Characteristics**	**Category**	**No.(%)**
Gender	Male	1302(68.9)
	Female	587(31.1)
Age (years)	<25	23(1.2)
	25 ~ 35	319(16.9)
	35 ~ 45	599(31.7)
	45 ~ 55	409(21.7)
	≥55	539(28.5)
Educational qualification	Secondary technical school and below	1564(82.8)
	Junior college	285(15.1)
	Bachelor	21(1.1)
	Master and above	19(1.0)
Title	No title	720(38.1)
	Junior title	844(44.7)
	Middle title	310(16.4)
	Senior title	15(0.8)
Work seniority	< 5	105(5.5)
	5-15	480(25.4)
	15-25	532(28.2)
	≥ 25	772(40.9)
Qualified to practice	Yes	1453(76.9)
	No	436(23.1)
Average income/years (ten thousand Yuan)	≤3	1451(76.8)
	3-5	315(16.7)
	5-8	99(5.2)
	≥ 8	24(1.3)
Individual income levels in the local*	The upper layer	12(0.6)
	Between upper and middle	57(3.0)
	Middle level	488(25.9)
	Between middle and lower	860(45.5)
	The lower layer	472(25.0)
Income satisfaction	Satisfied	113(6.0)
	Moderate (not too bad)	725(38.4)
	Dissatisfied	1051(55.6)

*The income levels are in relation to town-wide income levels.

### Turnover intention of village doctors

The results of the investigation conducted in Xiangyang City showed that 63.2% of the village doctors had no plans of leaving where they are currently employed, but more than one-third (36.8%) of village doctors considered leaving.

### Degree of job satisfaction of village doctors

The statistical results of the job satisfaction degree scores of the investigated village doctors indicated that village doctors were more satisfied with *the competence of my manager in making decisions* (2.87 ± 0.815), *the chance to do something that makes use of my abilities* (2.81 ± 0.768), *the way my boss handles his\her workers* (2.76 ± 0.856), and *been able to keep busy and fulfilling* (2.73 ± 0.818). They were most dissatisfied with *my pay and the amount of work I do* (1.99 ± 1.092), *the chances for advancement on this job* (2.21 ± 0.911), and *the work conditions* (2.34 ± 0.987).

Table 2 showed the respondent characteristics of the village doctors in the study area. A score of more than 2 in each component of job satisfaction were classified as the “satisfied” group. A score of “2” represented moderate (not too bad); the rest were “dissatisfied”.

**Table 2:** **Job satisfaction degree of the investigated village doctors**

**Option**	**Satisfactory n(%)**	**Not too bad n(%)**	**Dissatisfactory n(%)**	**Mean ± SD**
1. Been able to keep busy and fulfilling	1324(70.1)	421(22.3)	144(7.6)	2.73 ± 0.818
2. Level of attention by leaders	1247(66.0)	487(25.8)	155(8.2)	2.71 ± 0.855
3. The way my boss handles his\her workers	1338(70.8)	397(21)	154(8.2)	2.76 ± 0.856
4. The competence of my manager in making decisions	1380(73.1)	403(21.3)	106(5.6)	2.87 ± 0.815
5. Work stability	1238(65.5)	511(27.1)	140(7.4)	2.71 ± 0.839
6. The chance to do something that makes use of my abilities	1330(70.4)	464(24.6)	95(5.0)	2.81 ± 0.768
7. The way organization policies are put into practice	1056(55.9)	618(32.7)	215(11.4)	2.53 ± 0.900
8. My pay and the amount of work I do	683(36.2)	560(29.6)	646(34.2)	1.99 ± 1.092
9. The chances for advancement on this job	694(36.7)	854(45.2)	341(18.1)	2.21 ± 0.911
10. The chance to try my own methods of doing the job	1071(56.7)	687(36.4)	131(6.9)	2.59 ± 0.796
11. The work conditions	929(49.2)	596(31.5)	364(19.3)	2.34 ± 0.987
12. The feeling of accomplishment I get from the job	1125(59.6)	579(30.6)	185(9.8)	2.58 ± 0.878

### Socio-demographic characteristics related to the turnover intention of village doctors

This study used chi-square test to determine the correlation between all indexes (including socio-demographic factors and work satisfaction degree) and the turnover intention of village doctors. A value 1 was set for village doctors who was “considering to leave the current employing organization” and 0 for those who “do not consider leaving their current employing organization”. The results showed that, among the socio-demographic and job satisfaction degree indexes of village doctors, individual income levels in the local, income satisfaction and job satisfaction degree were related to village doctor turnover intention (p < 0.05) (Table 3).

**Table 3:** **Univariate analyses examing socio-demographic factors associated with the turnover intention of the village doctors**

**Indexes**	**With turnover intention n(%)**	**Without turnover intention n(%)**	**χ** ^**2**^	**p**
**Gender**				
Male	483(25.6)	819 (43.3)		
Female	212(11.2)	375(19.9)	0.167	0.682
**Educational qualification**				
Secondary technical school and below	577(30.5)	987(52.3)		
Junior college	104(5.5)	181(9.6)		
Bachelor	8(0.4)	13(0.7)		
Master and above	6(0.3)	13(0.7)	0.255	0.968
**Work seniority**				
< 5	30(1.6)	75(4.0)		
5-15	193(10.2)	287(15.2)		
15-25	196(10.4)	336(17.8)		
≥ 25	276(14.6)	496(26.2)	5.820	0.121
**Qualified to practice**				
Yes	528(28.0)	925(49.0)		
No	167(8.8)	269(14.2)	0.556	0.456
**Individual income levels in the local**				
The upper layer		4(0.2)	8(0.4)	
Between upper and middle	15(0.8)	42(2.2)		
Middle level	134(7.1)	354(18.7)		
Between middle and lower	320(16.9)	540(28.6)		
The lower layer	222(11.8)	250(13.3)	42.385	**<0.001**
**Income satisfaction**				
Satisfactory	18(0.9)	95(5.0)		
Moderate (not too bad)	177(9.4)	548(29.0)		
Dissatisfactory	500(26.5)	551(29.2)	121.452	**<0.001**

Bold values (p < 0.05) are statistically significant.

### Job satisfaction and the turnover intention of village doctors

Table 4 showed the existence of significant associations between job satisfaction and the turnover intention of village doctors. The components of job satisfaction, including *been able to keep busy and fulfilling (χ*^2^ *= 63.259), level of attention by leaders (χ*^*2*^ *= 94.244), the way my boss handles his\her workers (χ*^*2*^ *= 107.351), the competence of my manager in making decisions (χ*^*2*^ *= 107.944), work stability (χ*^*2*^ *= 148.266), the chance to do something that makes use of my abilities (χ*^*2*^ *= 84.353), the way organization policies are put into practice (χ*^*2*^ *= 145.606), my pay and the amount of work I do (χ*^*2*^ *= 140.592), the chances for advancement on this job (χ*^*2*^ *= 113.797), the chance to try my own methods of doing the job(χ*^*2*^ *= 46.986), the work conditions (χ*^*2*^ *= 159.456), the feeling of accomplishment I get from the job (χ*^*2*^ *= 97.266)* had significant associations with intention to leave (P < 0.001).

**Table 4:** **Univariate analyses examing job satisfaction associated with the turnover intention of the village doctors**

**Job satisfaction**	**Turnover intention**			
**Component**	**Yes n(%)**	**No n(%)**	**χ** ^**2**^	**p**
1. Been able to keep busy and fulfilling				
Dissatisfied	89(4.7)	55(2.9)		
Not too bad	186(9.8)	235(12.5)		
Satisfied	420(22.2)	904(47.9)	63.259	<0.001
2. level of attention by leaders				
Dissatisfied	103(5.5)	52(2.8)		
Not too bad	216(11.4)	271(14.3)		
Satisfied	376(19.9)	871(46.1)	94.244	<0.001
3. The way my boss handles his\her workers				
Dissatisfied	107(5.7)	47(2.5)		
Not too bad	181(9.6)	216(11.4)		
Satisfied	407(21.5)	931(49.3)	107.351	<0.001
4. The competence of my manager in making decisions				
Dissatisfied	82(4.3)	24(1.3)		
Not too bad	184(9.7)	219(11.6)		
Satisfied	429(22.7)	951(50.4)	107.944	<0.001
5. Work stability				
Dissatisfied	110(5.8)	30(1.6)		
Not too bad	225(11.9)	286(15.1)		
Satisfied	360(19.1)	878(46.5)	148.266	<0.001
6. The chance to do something that makes use of my abilities				
Dissatisfied	68(3.6)	27(1.4)		
Not too bad	213(11.3)	251(13.3)		
Satisfied	414(21.9)	916(48.5)	84.353	<0.001
7. The way organization policies are put into practice				
Dissatisfied	148(7.8)	67(3.5)		
Not too bad	261(13.8)	357(18.9)		
Satisfied	286(15.2)	770(40.8)	145.606	<0.001
8. My pay and the amount of work I do				
Dissatisfied	355(18.8)	291(15.4)		
Not too bad	163(8.6)	397(21.0)		
Satisfied	177(9.4)	506(26.8)	140.592	<0.001
9. The chances for advancement on this job				
Dissatisfied	209(11.1)	132(7.0)		
Not too bad	292(15.4)	562(29.7)		
Satisfied	194(10.3)	500(26.5)	113.797	<0.001
10. The chance to try my own methods of doing the job				
Dissatisfied	79(4.2)	52(2.8)		
Not too bad	277(14.7)	410(21.7)		
Satisfied	339(17.9)	732(38.7)	46.986	<0.001
11. The work conditions				
Dissatisfied	231(12.2)	133(7.0)		
Not too bad	224(11.9)	372(19.7)		
Satisfied	240(12.7)	689(36.5)	159.456	<0.001
12. The feeling of accomplishment I get from the job				
Dissatisfied	123(6.5)	62(3.3)		
Not too bad	236(12.5)	343(18.1)		
Satisfied	336(17.8)	789(41.8)	97.266	<0.001

### Analysis of the multiple factors that influence the turnover intention of village doctors

The binary logistic regression method was used to analyze the factors that influence the turnover intention of village doctors. We analyzed the significantly related factors (p < 0.05) obtained from a previous study. *Income satisfaction, the way organization policies are put into practice, my pay and the amount of work I do, the chances for advancement on this job*, and *the work conditions* were significantly related to the turnover intention of village doctors. The turnover intention of village doctors who were satisfied with their income was lower and 0.284 times lower than that of the doctors who were dissatisfied with their income. (OR = 0.284, 95%CI = 0.161–0.501, p = <0.001). The turnover intention of village doctors who were dissatisfied with *the way organization policies are put into practice* was 1.735 times higher that of others who were satisfied with it (OR = 1.735, 95%CI = 1.121–2.687, p = 0.013). Compared with the village doctors who were satisfied with their work conditions, the turnover intention of village doctor who were dissatisfied with their work conditions was 2.406 times higher (OR = 2.406, 95%CI = 1.686–3.435, p <0.001) (Table 5). This model had goodness-of-fit under the Hosmer–Lemeshow test (χ^2^ = 28.462, P < 0.001).

**Table 5:** **Multivariable analyses examing factors associated with the turnover intention of village doctors**

	**B**	**P**	**OR**	**95%CI**
**Individual income levels in the local**		0.560		
The upper layer	−0.161	0.812	0.851	(0.226, 3.207)
Between upper and middle	−0.391	0.267	0.676	(0.339, 1.349)
Middle level	−0.238	0.138	0.788	(0.576, 1.080)
Between middle and lower	−0.168	0.199	0.845	(0.653, 1.093)
The lower layer				
**Income satisfaction**		**<0.001**		
Satisfied	−1.259	<0.001	0.284	(0.161, 0.501)
Moderate (not too bad)	−0.623	<0.001	0.536	(0.418, 0.689)
Dissatisfied				
**Been able to keep busy and fulfilling**		0.818		
Dissatisfied	−0.144	0.537	0.866	(0.547, 1.369)
Not too bad	−0.007	0.963	0.993	(0.739, 1.334)
**Level of attention by leaders**		0.654		
Dissatisfied	0.148	0.587	1.159	(0.681, 1.973)
Not too bad	0.150	0.371	1.161	(0.837, 1.612)
**The way my boss handles his/her workers**		0.642		
Dissatisfied	0.256	0.363	1.292	(0.743, 2.247)
Not too bad	0.101	0.575	1.106	(0.778, 1.573)
**The competence of my manager in making decisions**		0.088		
Dissatisfied	0.701	0.030	2.017	(1.068, 3.807)
Not too bad	0.163	0.320	1.177	(0.854, 1.621)
**Work stability**		0.055		
Dissatisfied	0.670	0.021	1.955	(1.107, 3.454)
Not too bad	0.210	0.176	1.233	(0.910, 1.671)
**The chance to do something that makes use of my abilities**		0.939		
Dissatisfied	0.049	0.875	1.050	(0.569, 1.939)
Not too bad	0.057	0.726	1.059	(0.769, 1.457)
**The way organization policies are put into practice**		**0.028**		
Dissatisfied	0.551	0.013	1.735	(1.121, 2.687)
Not too bad	0.294	0.050	1.342	(1.000, 1.801)
**My pay and the amount of work I do**		**<0.001**		
Dissatisfied	0.166	0.352	1.180	(0.832, 1.674)
Not too bad	−0.433	0.014	0.648	(0.459, 0.916)
**The chances for advancement on this job**		**0.011**		
Dissatisfied	−0.076	0.723	0.927	(0.609, 1.410)
Not too bad	−0.422	0.010	0.655	(0.475, 0.905)
**The chance to try my own methods of doing the job**		0.086		
Dissatisfied	−0.580	0.027	0.560	(0.335, 0.936)
Not too bad	−0.097	0.541	0.907	(0.678, 1.215)
**The work conditions**		**<0.001**		
Dissatisfied	0.878	<0.001	2.406	(1.686, 3.435)
Not too bad	0.472	0.003	1.604	(1.172, 2.194)
**The feeling of accomplishment I get from the job**		0.531		
Dissatisfied	0.228	0.316	1.256	(0.804, 1.961)
Not too bad	−0.019	0.898	0.981	(0.736, 1.308)
**Constant**	−0.563	**0.000**	0.569	

Bold values (p < 0.05) are statistically significant.

## Discussion

Currently, there are many problems concerning the village doctors. Most village doctors in China lack security insurance, such as pension insurance. Problems such as low education levels, lower income, work pressure, and turnover intention are more prominent [3,20,21]. These problems still have not been resolved. This is why some of the trained village doctors abandoned medicine for agriculture or business [22].

Village doctors are the neglected group among doctors, similar to family physicians in some countries, especially in the developing countries. Being a family physician elicits a response that conveys disappointment as if to say, “Oh! You’re just a family physician!” [23].

The village doctors were trained using minimum funds to solve the most difficult healthcare problems of the peasants, who accounted for the largest proportion of the population [5]. The development goal of village doctors is to meet the basic health service demands of people in rural areas.

The village doctors in China, as well as the strategy development model, will have an important impact on the development of human resources on health care in rural areas, especially for developing countries. The further development and the establishment of the functions and status of rural doctors are significant in the process of establishing a basic medical system in China. However, this study shows that more than one-third of village doctors (36.8%) claimed that they had the intention to resign. The turnover intention of village doctors is a serious problem and calls for more attention. Similarly, a Canada study found that stress and burnout were related to the desire to give up practice among Canadian family physicians [24].

The survey of Mohr et al. shows that positive linear trends were observed for pay satisfaction, management of achievement, skill development, workplace civility, and satisfaction with senior management [25]. According to Mosadeghrad et al., job stress is a serious threat to the work life quality of healthcare employees and can cause hostility, aggression, absenteeism, and turnover [26]. Primary care physicians’ retention in rural areas is related to physicians’ practice situations, economies and their satisfaction [27,28].

This study shows that among the investigated factors of village doctors in China, individual income levels in the local, income satisfaction, and job satisfaction are the factors that influence the turnover intention of village doctors. This result is similar to O’Neill et al.’s [29] survey of medical workers, wherein turnover intention has a significant negative correlation with the degree of work satisfaction and organization identification. Similarly, Research has shown that physician dissatisfaction is associated with an intention to leave a practice [30-32]. In particular, physician satisfaction with employers has been shown to be negatively correlated with intention to leave a practice [33].

Behmann et al.’s survey of job satisfaction among primary care physicians shows that the greatest degree of dissatisfaction by some margin was expressed with regard to administrative tasks [34]. In our study, village doctors were more satisfied with *the competence of my manager in making decisions* (2.87 ± 0.815), *the chance to do something that makes use of my abilities* (2.81 ± 0.768), *the way my boss handles his\her workers* (2.76 ± 0.856), and *been able to keep busy and fulfilling* (2.73 ± 0.818). By contrast, they were most dissatisfied with *my pay and the amount of work I do* (1.99 ± 1.092), *the chances for advancement on this job* (2.21 ± 0.911), and *the work conditions* (2.34 ± 0.987). In our survey, in terms of working time, village doctors worked 6.5 days a week on average, with 9.97 hours a day. About 63% of the respondents worked seven days a week, and 86.8% of the respondents should work overtime during the holidays. These findings indicate that village doctors believe that their current remuneration does not match the pressure and the amount of their work. Village doctors lack suitable opportunities for promotion and work conditions. On the one hand, people who obtained licensed certificate for village doctors can only work in the village medical institutions. On the other hand, village doctors’ opportunities for continuing education and training are insufficient. The lack of chances for advancement in work is an important reason that leads to village doctors’ turnover intention. Compared with the doctors in the city, the work conditions of village doctors are tough, such as simple and crude medical equipment, especially in many poor areas. In addition, the village doctor is incorporated into the social pension insurance system in rural areas as the farmer identity. The level of compensation is very low and unfair to village doctors. Through the research of village doctors’ income and pension status, we found that it is difficult for them to achieve guaranteed pension under the existing system and income status. Therefore, it is necessary to promote the system of pension which is specific to village doctors in nationwide.

Job satisfaction reduces employee turnover, absenteeism, and the number of thefts at work, which in turn reduces organizational costs [35]. Turnover intention can be avoided by increasing the degree of job satisfaction because job satisfaction has a negative correlation with intention to leave [36]. Therefore, the results of the investigation on the degree of work satisfaction and turnover intention of village doctors indicate that the government should raise the income of village doctors and provide them with suitable promotion opportunities and security insurance, such as health insurance and pension insurance. In addition, low education levels of village doctors, as a barrier to achieve the educational goals for the village doctors set by the government, should also be tackled through further on the-job training or by recruiting more college medical graduates into the village clinics [3]. In order to promote the further education of village doctors and reasonable subsidy plans formulated for village doctors, the effective incentive mechanism should be built in time. The technical and labor value of village doctors should be respected.

## Conclusion

The results of the investigation conducted in Xiangyang City showed that village doctors were most dissatisfied with *my pay and the amount of work I do* (1.99 ± 1.092), *the chances for advancement on this job* (2.21 ± 0.911), and *the work conditions* (2.34 ± 0.987). Approximately 36.8% of village doctors considered leaving the organization where they were currently employed. *Income satisfaction*, *the way organization policies are put into practice*, *my pay and the amount of work I do*, *the chances for advancement on this job* and *the work conditions* were significantly related to the turnover intention of village doctors.

The findings may be of interest to policy makers in health department considering the consequences of the current the shortage of village doctors. This study highlighted the influence of job satisfaction on turnover intention of village doctors who devote themselves to health care services in the rural areas.

## References

[1] Li X, Chongsuvivatwong V, Xia X, Sangsupawanich P, Zheng W, Ma K (2012). Revisiting current “barefoot doctors” in border areas of China: system of services, financial issue and clinical practice prior to introducing integrated management of childhood. BMC Public Health.

[2] Zhang D, Unschuld PU (2008). China’s barefoot doctor: past, present, and future. Lancet.

[3] Xu H, Zhang W, Zhang X, Qu Z, Wang X, Sa Z, Li Y, Zhao S, Qi X, Tian D (2013). Longitudinal study of rural health workforce in five counties in China: research design and baseline description. Hum Resour Health.

[4] Wen C (1975). Barefoot doctors in China. Nurs Dig.

[5] Zhang RX, Zhang W (2009). The rise and demise of the Chinese barefoot doctor. Zhonghua Yi Shi Za Zhi.

[6] Ministry of Health (2012). 2012 China Health Statistics Yearbook (In Chinese).

[7] Wang C, Rao K, Wu S, Liu Q (2013). Health care in China: improvement, challenges, and reform. Chest.

[8] Ding Y, Smith HJ, Fei Y, Xu B, Nie S, Yan W, Diwan VK, Rainer S (2013). Factors influencing the provision of public health services by village doctors in Hubei and Jiangxi provinces. China Bull World Health Organ.

[9] Shi L, Hung LM, Song K, Sarika R, Jenna T, Sun X, Li H, Meng Q (2013). Chinese primary care physicians and work attitudes. Int J Health Serv.

[10] Colwill JM, Cultice JM, Kruse RL (2008). Will generalist physician supply meet demands of an increasing and aging population?. Health Aff (Millwood).

[11] Hann M, Gravelle H (2004). The maldistribution of general practitioners in England and Wales: 1974–2003. Br J Gen Pract.

[12] Yip WC, Hsiao WC, Chen W, Hu S, Ma J, Maynard A (2012). Early appraisal of China’s huge and complex health-care reforms. Lancet.

[13] Chen L, Evans T, Anand S, Boufford JI, Brown H, Chowdhury M, Cueto M, Dare L, Dussault G, Elzinga G, Fee E, Habte D, Hanvoravongchai P, Jacobs M, Kurowski C, Michael S, Pablos-Mendez A, Sewankambo N, Solimano G, Stilwell B, de Waal A, Wibulpolprasert S (2004). Human resources for health: overcoming the crisis. Lancet.

[14] Anand S, Barnighausen T (2004). Human resources and health outcomes: cross-country econometric study. Lancet.

[15] Zhang Y, Feng X (2011). The relationship between job satisfaction, burnout, and turnover intention among physicians from urban state-owned medical institutions in Hubei, China: a cross-sactional study. BMC Health Ses.

[16] March JG, Simon HA (1958). Organizations.

[17] Mobley WH, William H (1977). Intermediate linkages in the relationship between job satisfaction and employee turnover. J Appl Psychol.

[18] Mosadeghrad AM, Ferlie E, Rosenberg D (2008). A study of the relationship between job satisfaction, organizational commitment and turnover intention among hospital employees. Health Serv Manage Res.

[19] Gauci Borda R, Norman I (1997). Factors influencing turnover and absence of nurses: a research review. Int J Nurs Stud.

[20] Wang J, Su J, Zuo H, Jia M, Zeng Z (2013). What interventions do rural doctors think will increase recruitment in rural areas: a survey of 2778 health workers in Beijing?. Hum Resour Health.

[21] Shi L, Song K, Rane S, Sun X, Li H, Meng Q (2013). Factors associated with job satisfaction by Chinese primary care providers. Primary Health Care Res Dev.

[22] Chen Z, Wang Y, Cui X, Sun M, Li C, Wang H, Zhong X, Zhang W, Sun X, Hao M (2009). The origin, development and status quo of rural doctors in China. Chinese Primary Health Care.

[23] Ladouceur R (2012). What has become of family physicians?. Can Fam Physician.

[24] Lee FJ, Stewart M, Brown JB (2008). Stress, burnout, and strategies for reducing them: What’s the situation among Canadian family physicians?. Can Fam Physician.

[25] Mohr DC, Bauer MS, Penfold RB (2013). Changes in VA psychiatrists’ attitudes about work environment and turnover during mental health service enhancement. Psychiatr Serv.

[26] Mosadeghrad AM, Ferlie E, Rosenberg D (2011). A study of relationship between job stress, quality of working life and turnover intention among hospital employees. Health Serv Manage Res.

[27] Cutchin MP, Norton JC, Quan MM, Bolt D, Hughes S, Lindeman B (1994). To stay or not to stay: issues in rural primary care physician retention in Eastern Kentucky. J Rural Health.

[28] Pathman DE, Konrad TR, Dann R, Koch G (2004). Retention of primary care physicians in rural health professional shortage areas. Am J Public Health.

[29] O’Neill JL, Gaither CA (2007). Investigating the relationship between the practice of pharmaceutical care, construed external image, organizational identification, and job turnover intention of community pharmacists. Res Soc Adm Pharm.

[30] Buchbinder SB, Wilson M, Melick CF, Powe NR (1999). Estimates of costs of primary care physician turnover. Am J Manag Care.

[31] Price JL, Mueller CW (1986). Absenteeism and Turnover of Hospital Employees.

[32] Pathman DE, Konrad TR, Williams ES, Scheckler WE, Linzer M, Douglas J (2002). Physician job satisfaction, job dissatisfaction, and physician turnover. J Fam Pract.

[33] Beasley JW, Karsh BT, Sainfort F, Hagenauer ME, Marchand L (2004). Quality of work life of family physicians in Wisconsin’s health care organizations: a WReN study. Wis Med J.

[34] Behmann M, Schmiemann G, Lingner H, Kuhne F, Hummers-Pradier E, Schneider N (2012). Job satisfaction among primary care physicians. : Results of a survey. Dtsch Arztebl Int.

[35] Greenberg J, Baron A (2000). Behavior in Organizations.

[36] Ching-I T, Yea-Ing LS, Hao-Yuan C (2007). Moderating effects of professional commitment on hospital nurses in Tai-wan original research article. J Prof Nurs.

